# The relationship between socio-demographic characteristics of patients and diagnostic delay in acute pulmonary thromboembolism

**DOI:** 10.3109/03009734.2010.530701

**Published:** 2011-02-11

**Authors:** Yilmaz Bulbul, Sibel Ayik, Funda Oztuna, Tevfik Ozlu, Sibel Sahin

**Affiliations:** Karadeniz Technical University, School of Medicine, Department of Chest Diseases, TrabzonTurkey

**Keywords:** Delay to diagnosis, delay to presentation, pulmonary embolism

## Abstract

**Background:**

In pulmonary thromboembolism (PE), delay to diagnosis is very common. In this study, we examined the role of patients and the socio-demographic characteristics in delayed diagnosis of PE.

**Patients and methods:**

We evaluated 156 PE patients for the dates of symptom onset, the dates of first visit to a health institution and diagnosis, signs and symptoms, and the socio-demographic characteristics. Delays were analyzed using the Mann-Whitney *U* test, and the predictors were analyzed using logistic regression analysis.

**Results:**

Of the patients, 60.3% visited a health institution within the first day of the symptoms. Mean time from symptoms to the first admission to a health institution (patient delay) was 2.04 ± 3.89 days (median 0 day, range 0–30). Current smoking, a high level of education, and co-morbidity were associated with longer patient delays. The time interval from first symptom to the diagnosis (total delay) was 7.93 ± 10.05 (median 4 days, range 0–45) days. While hypotension, syncope, and previous surgery/trauma were significantly associated with a shorter total delay, a previous visit to any health institution was associated with longer total delay.

**Conclusion:**

In conclusion, although some socio-demographic characteristics of patients such as smoking, educational status, and co-morbid diseases were found to be associated with delayed visit to any health institution, our results showed that physician or health system delays were more prominent in delayed diagnosis of PE.

## Introduction

Pulmonary thromboembolism (PE) is usually considered to be an acute disease. However, because of the non-specific nature of the signs and symptoms of the disease, delays from the onset of symptoms to hospital admission and to diagnosis are very common. Median time from symptoms to hospital admission was reported to vary between 4 and 7 days (1,2). In those studies, delays were reported to be associated with the severity of the disease (presence of multiple signs and symptoms, hypotension, or syncope) and the type of risk factors causing PE (1–4). However, most previous studies were designed retrospectively, and the potential role of patients was not studied. The primary aim of this study was to investigate the role of patients and socio-demographic characteristics in delayed visit to a health institution and in delayed diagnosis of PE.

## Materials and methods

### Study design

This prospective and observational study was conducted at Farabi Hospital, a tertiary care hospital with 750-bed capacity, at Karadeniz Technical University, Turkey, and was approved by the local ethics committee.

### Study setting and population

The study group comprised all adult patients with PE who were diagnosed and followed up by the Chest Clinic of Farabi Hospital between January 2007 and December 2008 and who agreed to participate in the study. Diagnosis of PE was confirmed using spiral Computed Tomography (CT) (Somatom volume zoom and sensation 16, Siemens, Erlangen, Germany) and perfusion scan (Siemens E-cam Dual-Head, USA). The date of symptom onset, the date of first visit to any health institution (health center or hospital), and the diagnosis were recorded. In addition to socio-demographic data (age, gender, education status, occupation, place of residence, and smoking status), the signs and symptoms (dyspnea, chest pain, hemoptysis, fever, deep vein thrombosis (DVT), syncope, and hypotension), co-morbid diseases, and risk factors of patients with PE were recorded. The time interval from the onset of symptoms to the first visit to any health institution (a health center or a hospital) was defined as ‘patient delay’, and the time interval from symptoms to the diagnosis was defined as ‘total delay’.

### Statistical analysis

The Mann-Whitney *U* test was used for the comparison of delays with socio-demographic characteristics, signs and symptoms, co-morbid conditions, and risk factors. Results are given as mean ± SD and median (range). *P* values <0.05 were considered to be significant. Factors that may be related to delays were analyzed using logistic regression.

## Results

During the study period, 200 patients with PE were diagnosed and followed up by our clinic. Of the total, 15 were excluded because of questionable date of symptom onset, and 29 were excluded because they were already hospitalized. Of the remaining 156 patients who participated in the study, 97 (62.2%) were females and 59 (37.8%) were males. Diagnosis was confirmed using spiral CT in 150 patients and lung scan in 6 patients. The mean age was 64.07 ± 15.90 years, and 87 of the patients (55.8%) were older than 65 years. Of the patients, 13.2% were currently smokers, 19.1% were ex-smokers, and 67.7% had never smoked. A total of 68 (43.8%) of the patients were living within a city center, and the remainder lived either in a small town (5.2%) or a village (51.0%). The proportion of patients who were from neighboring cities was 51.8%. Of the patients 59.6% were illiterate, and the remainder had graduated from either primary school (31.1%) or high school and university (9.3%). The majority of patients stated their occupation as housewife (58.4%), followed by farmer (13.6%) and others (worker, official, etc.). Altogether 132 patients (84.6%) had at least one co-morbid disease (cardiovascular diseases 51.2%, malignant diseases 8.3%, pulmonary disease 4.5%, endocrine diseases 3.8%, etc.). Of the patients, 30.6% had previous surgery or trauma, and 69.4% had medical risk factors for PE. Symptoms commonly seen at first presentation were dyspnea (73.1%), pleuritic chest pain (51.3%), syncope (16.0%), and hemoptysis (10.3%). The rate of hypotension was 15.5%, and the signs and symptoms of deep vein thrombosis were detected in 15%.

### Patient delay

After the onset of symptoms, the average delay from symptoms to the visit to a health institution was 2.04 ± 3.89 days (median 0 day, range 0–30). This interval was 4.25 ± 6.74 days (median 2.5 days, range 0–30) in current smokers and 1.74 ± 3.20 days (median 0 day, range 0–30) in those who had never smoked (*P* = 0.011). After the start of symptoms, patients with high educational status were admitted within 3.04 ± 5.29 days (median 0 day, range 0–30); however, patients with a lower educational status were admitted within 1.34 ± 2.42 days (median 0 day, range 0–11) (*P* = 0.028). While patients with a co-morbid disease were admitted within 2.25 ± 4.10 days (median 0 day, range 0–30), patients with no co-morbid condition were admitted to a health institution within 0.83 ± 2.09 days (median 0 day, range 0–7) (*P* = 0.021). Other socio-demographic and clinical factors were not found to be significantly associated with the patient delay.

Among the clinical and socio-demographical factors, univariate logistic regression analysis showed that only current smoking and education were the factors predicting delayed first visit to a health institution (Table I).

**Table I.** Univariate logistic regression analysis of clinical and socio-demographical factors for patient delay in pulmonary thromboembolism.

	Delayed presentation (after 24th hour)		
Variable	OR	95.0% CI	*P*
Age > 65 y	1.322	0.693–2.521	0.396
Gender	1.192	0.617–2.395	0.601
Education status (literacy)	2.036	1.043–3.974	0.037^a^
Occupation	0.903	0.627–1.302	0.585
Residence (in urban)	1.027	0.536–1.965	0.937
Current smoking	3.456	1.266–9.434	0.015^a^
Risk factor (surgery or trauma)	0.056	0.260–1.188	0.129
Symptoms and signs			
Dyspnea	1.099	0.532–2.273	0.798
Pain	0.918	0.483–1.744	0.795
Hemoptysis	1.593	0.564–4.494	0.379
Syncope	0.423	0.159–1.128	0.086
Hypotension	0.569	0.221–1.466	0.243
Deep vein thrombosis signs	1.368	0.470–3.986	0.566

^a^ Statistically significant.

### Total delay

Among 156 patients, 94 (60.3%) were admitted to a health institution within the first day of the symptoms; however, only 31 (19.9%) received final diagnosis of PE within the first 24 hours. Total delay across the group was 7.93 ± 10.05 days (median 4 days, range 0–45). There was no correlation between socio-demographic factors and delayed diagnosis (total delay) of PE. However, total delay was significantly longer in patients who were previously admitted to any health institution (7 days, range 0–45 versus 2 days, range 0–30; *P* < 0.001). Univariate logistic regression analysis showed that previous hospital or doctor visits were associated with an approximately 11 times longer diagnostic delay than the patients who did not visit a doctor or a hospital (Table II). On the contrary, patients were diagnosed earlier with a previous surgery/trauma (2 days, range 0–30 versus 4 days, range 0–45; *P* = 0.001), syncope (1 day, range 0–15 versus 4 days, range 0–45; *P* < 0.006), and hypotension (1 day, range 0–30 versus 4 days, range 0–45; *P* = 0.004). Logistic regression analysis also indicated a clear association between early diagnosis and previous surgery/trauma, syncope, and hypotension (Table II). No statistically significant association was found between total delay and the remaining socio-demographic and clinical factors.

**Table II.** Univariate logistic regression analysis of factors predicting delayed diagnosis (total delay) of pulmonary thromboembolism.

	Delayed diagnosis (after 24th hour)		
Variable	OR	95.0% CI	*P*
Age > 65 y	1.015	0.988–1.042	0.286
Gender	0.614	0.261–1.442	0.262
Education status (literacy)	2.805	1.123–7.008	0.027
Occupation	0.442	0.183–1.068	0.070
Residence (in urban)	0.677	0.307–1.491	0.333
Current smoking	1.722	0.465–6.378	0.416
Risk factor (surgery or trauma)	0.259	0.113–0.592	0.001
Symptoms and signs			
Dyspnea	1.140	0.477–2.726	0.767
Pain	1.358	0.617–2.992	0.447
Syncope	0.286	0.113–0.723	0.008^a^
Hypotension	0.267	0.105–0.682	0.006^a^
Deep vein thrombosis signs	1.683	0.359–7.880	0.509
Previous visits to a health institution	11.592	3.816–35.218	< 0.001^a^

^a^ Statistically significant.

### Delays and mortality

In-hospital mortality was 9.6% (15 patients) within the whole group. Of the patients who died, 14 visited a health institution within the first day of symptoms, and 1 patient was admitted the following day (*P* = 0.013). However, total delay and patient delay were not significantly different between patients who died and those who survived. Mortality rates were higher in patients with hypotension (25.0% versus 6.1%; *P* = 0.010) and those with a medical risk factor (15.0% versus 0%; *P* = 0.030).

## Discussion

PE is usually considered to be an acute illness. However, because the signs and symptoms are not specific, patients may experience significant delays from the onset of symptoms to presentation or diagnosis. In previous studies, the mean time from symptoms to presentation was reported to be 2.9–8.4 days, and mean time to diagnosis was 0.9–2.4 days (2,4,5). Timmons et al. reported that approximately 50% of patients presented within 24 hours of symptom onset (6). In other studies, 18.0%–30.4% of the patients were found to have visited a doctor/hospital after 1 week of symptom onset (2,5,6). In the current study, 60% of the patients were admitted to a health institution within the first 24 hours; however, less than half of them were diagnosed with PE within the same day.

Previous studies have investigated some clinical and demographic factors and their association with delay to presentation and to diagnosis. In a previous study, we reported that patients with more severe disease (presence of hypotension and tachypnea) had presented earlier (2). Ageno et al. also reported that the severity of the presentation (presence of multiple signs and symptoms) was associated with earlier diagnosis (3). In a recent study by Ozsu et al., it was reported that patients who had a surgical risk factor for PE and syncope were diagnosed earlier (4). The role of age, gender, co-morbidity, symptoms, and the location of thrombi in the pulmonary arterial tree were also investigated in the above-mentioned studies; however, the role of physicians, the role of patients, and socio-demographic factors were not studied.

Patient delay, the time interval from symptoms to first visit to a health institution, was 2 days. Interestingly, there was a reverse association between patient delay and educational status. Although the role of education in delayed diagnosis of PE was not investigated in previous studies, there are several studies reporting a clear association between education and early presentation/diagnosis of some other diseases such as malignancy and tuberculosis (7–9). Patient delay was also longer in current smokers. We think this delay was related to accompanying pulmonary symptoms and diseases due to smoking. In several studies, smoking has been shown to be well correlated with delay in seeking medical help (10,11). Contrary to previous studies, we found that presence of a co-morbid disease was clearly associated with patient delay (2–4). We think this was mostly related to the design of our study, because we analyzed delays as patient delay and total delay, separately. Indeed, while total delay was not affected by the presence of a co-morbid disease, patient delay was associated with co-morbid diseases.

Total delay, the average time from the start of symptoms to diagnosis, was 7.9 days. As we mentioned above, of this duration, only 2 days were related to patients themselves. We think the remaining 5.9 days were mostly related to the health system and the physicians. Actually, logistic regression analysis showed that previous hospital or doctor visits were associated with an approximately 11 times longer diagnostic delay. It is known that, because of the non-specific signs and symptoms of the disease, PE is usually underdiagnosed. It was reported that PE was confirmed *ante mortem* in only 30% of patients, with the remaining two-thirds diagnosed by autopsy (12,13). In the current study, we also confirmed that the presence of syncope, hypotension, or a prior trauma/surgery shortened the total delay. In an autopsy series, Goldhaber et al. reported that the accuracy of PE was far greater in postoperative patients (13). Ozsu et al. also reported that patients with a surgical risk factor were diagnosed earlier (4).

In the current study, no association was found between the length of delay and the mortality rate. Due to rapid technological advances in CT technology, spiral thorax CT is increasingly employed in the diagnosis of PE, and the diagnostic rate of embolism has increased (14). However, although the diagnosis of PE has increased with the use of CT and the associated use of enoxaparin has risen, a parallel reduction in mortality rates has not been reported (4).

The present study has a number of limitations. Firstly, we detected that an important part of delay was associated with the health system and previous doctor visits. Therefore, it might be more helpful to investigate system- and physician-related factors. Again, our results would be more valuable if we were able to record alternative diagnoses proposed for patients on their first visit to a health institution.

We conclude that there was a considerable patient delay from the onset of PE symptoms until the first visit to a health institution and diagnosis. In addition to the socio-demographic characteristics, including smoking status and high educational status, the presence of a co-morbid disease was also associated with patient delay in PE. However health system- and physician-related delays were more prominent, and a previous visit to a health institution was associated with approximately 11 times longer delay in diagnosis of PE.

## References

[1] Jimenez Castro D, Sueiro A, Diaz G, Escobar C, Garcia-Rull S, Picher J (2007). Prognostic significance of delays in diagnosis of pulmonary embolism. Thromb Res.

[2] Bulbul Y, Ozsu S, Kosucu P, Oztuna F, Ozlu T, Topbaş M (2009). Time delay between onset of symptoms and diagnosis in pulmonary thromboembolism. Respiration.

[3] Ageno W, Agnelli G, Imberti D, Moia M, Palareti G, Pistelli R (2008). Factors associated with the timing of diagnosis of venous thromboembolism: Results from the MASTER registry. Thromb Res.

[4] Ozsu S, Oztuna F, Bulbul Y, Topbas M, Ozlu T, Kosucu P (2010). The role of risk factors in delayed diagnosis of pulmonary embolism. Am J Emerg Med.

[5] Elliott CG, Goldhaber SZ, Jensen RL (2005). Delays in diagnosis of deep vein thrombosis and pulmonary embolism. Chest.

[6] Timmons S, Kingston M, Hussain M, Kelly H, Liston R (2003). Pulmonary embolism: differences in presentation between older and younger patients. Age Ageing.

[7] Storla DG, Yimer S, Bjune GA (2008). A systematic review of delay in the diagnosis and treatment of tuberculosis. BMC Public Health.

[8] Abdel-Fattah MM, Anwar MA, Mar1 E, El-Shazly MK, Zaki AA, Bedwani RN (1999). Patient and system related diagnostic delay in breast cancer. Eur J Public Health.

[9] Vineis P, Fornero G, Magnino A, Giacometti R, Ciccone G (1993). Diagnostic delay, clinical stage, and social class: a hospital based study. J Epidemiol Community Health.

[10] Basnet R, Hinderaker SG, Enarson D, Malla P, Mørkve O (2009). Delay in the diagnosis of tuberculosis in Nepal. BMC Public Health.

[11] Hansen RP, Olesen F, Sørensen HT, Sokolowski I, Søndergaard I (2008). Socioeconomic patient characteristics predict delay in cancer diagnosis: a Danish cohort study. BMC Health Serv Res.

[12] Kroegel C (2003). Advances in the diagnosis and treatment of pulmonary embolism. Pulmonary embolism—how can you mend a broken clot?. Respiration.

[13] Goldhaber SZ, Hennekens CH, Evans DA, Newton EC, Godleski JJ (1982). Factors associated with correct antemortem diagnosis of major pulmonary embolism. Am J Med.

[14] Kroegel C, Reissig A (2004). Computed tomography imaging in pulmonary embolism—the other side of medal. Respiration.

